# Salutogenesis and Coping: Ways to Overcome Stress and Conflict

**DOI:** 10.3390/ijerph17186667

**Published:** 2020-09-13

**Authors:** Orna Braun-Lewensohn, Claude-Hélène Mayer

**Affiliations:** 1 Department of Interdisciplinary Studies, Conflict Management & Resolution Program, Ben-Gurion University of the Negev, Beer-Sheva 8410501, Israel; 2 Department of Industrial Psychology and People Management, Auckland Park Campus, University of Johannesburg, Johannesburg 2006, South Africa; claudemayer@gmx.net; 3 Institut für Therapeutische Kommunikation und Sprachgebrauch, Europa Universität Viadrina, Logenstrasse 11, 15230 Frankfurt (Oder), Germany

**Keywords:** salutogenesis, stress, coping, conflict

## Abstract

This Special Issue aims to explore the concepts of stress, coping resources, and coping strategies, which are rooted in several theories, such as the stress and coping theory and the salutogenesis theory, and to understand how their core constructs are manifested in various ethnic and cultural groups around the world. This Special Issue includes 13 articles on salutogenesis and coping from different disciplinary, socio-cultural, historical, political, and economic perspectives. These articles address salutogenesis on the individual, organizational, and societal levels. The empirical studies are based in different societal and national contexts and refer to different ethnic groups within those contexts. Other studies examine international leaders in industry from a global perspective and present a systemic review of the literature concerning individuals in specific professions, such as nursing. The studies in the current Special Issue set the ground for continuing research toward even more comprehensive theoretical grounds; studies that incorporate several theoretical backgrounds and explore a broad theoretical model that may help us to understand successful adaptation in various contexts. In summary, results of studies that incorporate these theories may promote our understanding of the effects of coping resources and strategies, including acculturation strategies used among minority groups for positive adaptation.

## 1. Introduction

The stress appraisal and coping theory [1], views coping as an interactional process between an individual and his/her environment, which can be defined as the effort exerted by the individual to deal with demands from the environment, in order to make those demands more tolerable and reduce stress and conflict. This means that the characteristics of an individual and the way that he or she appraises a situation are important elements for that individual’s well-being in the aftermath of a stressful or conflictual encounter. Moreover, in the cognitive process of appraisal, one of the components that the individual assesses is the resources s/he has to deal with the situation.

To this end, sense of coherence (SOC), which is the central component of the salutogenic model, can be perceived as a secondary appraisal that facilitates the exploration of resources available to the individual to deal with the stressful situation. The salutogenic model looks for functions of positive qualities rather than healing from sickness [2,3]. Its main construct, SOC, is an enduring tendency to see the world as more or less comprehensible, manageable, and meaningful [4]. In accordance with salutogenesis, a person with a strong SOC is more likely to evaluate a stimulus as neutral [2]. Therefore, an individual with a strong SOC is less likely than one with a weak SOC to perceive stressful situations as threatening and, therefore, as anxiety-provoking. SOC determines the ability of individuals to use resources that are available to them to promote their well-being [5]. Moreover, SOC includes components that consolidate resilience and expand subjective mental health [2].

Coping strategies are the behavioral component of the process and can be defined as the actual effort made in the attempt to render a perceived stressor or conflict more tolerable and to minimize the distress induced by the situation. Most models of coping assume that individuals who cope more effectively with stressful and conflictual life events will exhibit lower levels of anxiety or depression [1]. Studies have shown that emotion-focused strategies of coping tend to be associated with more psychological problems, whereas, problem-focused strategies or active coping tend to be linked to more well-being [6].

We thought that looking at the stress appraisal and coping theory of Lazarus and Folkman [1] and the salutogenesis model of Antonovsky together might provide us with a more comprehensive understanding of the resources that facilitate certain coping strategies and the behaviors that are more or less adaptive in different situations of stress and conflict. Through the lens of a more integrative model, several issues can be highlighted. First, different types of events can be examined to determine whether different resources, coping, and responses are exhibited. Second, cultural contexts can be taken into consideration to understand the cognitive, behavioral, and emotional processes of individuals in the course of these events. Finally, we can consider a more comprehensive set of outcomes that includes positive (and not only pathological) outcomes.

## 2. The Aim of this Special Issue

This Special Issue aims to explore the concepts of stress, coping resources, and coping strategies, which are rooted in several theories, such as the stress and coping theory of Lazarus and Folkman [1], and the salutogenesis theory of Antonovsky [7], and to understand how their core constructs are manifested in various ethnic and cultural groups around the world.

These theories suggest that their main concepts, namely, several ways of coping, hope, personal and collective SOC, and others, are universal and, therefore, predict that, in all cultures, they could be considered as potential protectors against stress. However, to date, studies involving a non-Western population have reported ambiguous results.

In this Special Issue, we aim to address these concerns comprehensively by inviting researchers from around the world to present their studies based on special research methods and mixed research methods. There studies will enable a fundamental understanding of positive adaptation in stressful and conflictual situations among various cultural and ethnic groups and in different contexts around the world.

## 3. The Contributions in this Special Issue

This Special Issue includes 13 articles on salutogenesis and coping from different disciplinary, socio-cultural, historical, political, and economic perspectives. These articles address salutogenesis on individual, organizational, and societal levels. The empirical studies are based in different societal and national contexts, including Israel, Spain, Norway, and the Democratic Republic of Congo, and refer to different ethnic groups within those countries. Other studies examine international leaders in industry from a global perspective and present a systemic review of the literature concerning individuals in specific professions, such as nursing.

We decided to organize this Special Issue around several themes: first, the age of the participants (from youngest to adults); second, special populations such as minority groups, volunteers, health workers etc.; and third, the settings on which the studies focused, for example, workplaces. The Special Issue opens with a paper on adolescents, the youngest group examined in this volume, and moves on to a paper on a minority student population. Other papers focused on minority groups highlight refugees from the civil war in Syria and educated ultra-orthodox Jews in the workplace. The last prominent theme of the current Special Issue is the workplace, a focal point of many of the articles in this volume. Two articles focus on professionals in the special context of political violence, while some others focus on work in health-care settings. Following these articles, one paper focuses on SOC and coping among employees in the Democratic Republic of Congo. Finally, the last article in this volume explores SOC among international leaders and compassionate love as a coping mechanism.

Here is a brief introduction to the articles that make up this Special Issue.

A highly interesting study explores the associations between sex, age, socio-economic status, stress, SOC, and health among adolescents in Norway. The authors, Unni Karin Moksnes and Geir Arild Espnes [8], investigate SOC and stress interrelationships and point out that SOC is a major coping resource in the context of depression and mental well-being.

Sarah Abu-Kaf and Enas Khalaf [9], present a study on experiences of acculturative stress among Arab students at Israeli institutions of higher learning. The authors combined the theories of coping and salutogenesis and report gender differences in the use of different coping strategies and in levels of depressive symptoms. Moreover, they report that SOC differentially mediates the relationships between acculturative stress and depressive symptoms.

Antony Bernard and Aden Paul Flotman [10], explore the identity work of a group of eight consulting psychology doctoral students. The students wrote self-reflective essays about becoming a consulting psychologist and findings describe how students cope with performance.

Orna Braun-Lewensohn, Sarah Abu-Kaf and Khaled Al-Said [11], present findings on the coping resources and mental health of women in refugee camps. These authors explore personal and the community SOC and their influence on perceived danger and coping. The authors also demonstrate that SOC is crucial for good adaptation. These results are discussed in light of salutogenic theory.

In her article on coping strategies of college-educated, ultra-orthodox Jews in the general Israeli workforce, Tehila Kalagy [12], speaks about societal transitions and the changes in the values of ethnic groups and in workplaces. This article contributes to minority research, as well as our understanding of the professional integration and adaptability of members of minority groups in the workplace and how those individuals cope with the challenges they face.

Tal Litvak-Hirsch and Alon Lazar [13] explore long-term mindfulness training and its contribution to personal and professional coping among teachers living in a conflict zone. These authors present their findings from a qualitative study conducted in the Western Negev region of Israel. Interviewees reported that their coping skills had been heightened as result of being able to put aside intrusive thoughts and feelings that used to paralyze them and focus on active coping, centered on what they needed to do promptly. The interviewees also reported increased compassion and self-acceptance of emotions and behaviors. This article presents an important contribution to stress management in war zones through mindfulness training.

In their article, Dorit Segal-Engelchin, Netta Achdut, Ephrat Huss and Orly Sarid [14], focus on CB-ART (cognitive behavioral and art-based) intervention during the 2014 Gaza conflict. The authors present findings regarding the ability of interventions to decrease stress and trauma among individuals working in medical professions. Specifically, they describe how arts-based methods supported coping and built resources to deal with stress and trauma.

The next article sheds light on the situation in South Africa and refers to the experiences of volunteers in the health-care context and their well-being. Antoni Barnard and Aleksandra Furtak [15], argue that volunteers in South Africa need psychological resilience from a salutogenic perspective. What really keeps them healthy is an inner drive and a calling in the context of the work orientation, which can be increased when organizations invest in developmental interventions.

Natura Colomer Pérez, Elena Chover-Sierra, Vicente Gea-Caballero and Joan J Paredes-Carbonell [16], address people’s health-assets mapping processes and design-dynamization strategies for health promotion. The authors present a salutogenic model of health and a health-assets model and report findings from the nursing context in Spain. Their results show that SOC can be strengthened through the use of salutogenic and asset-based approaches.

The article by Giuseppe Michele Masanotti, Silvia Paolucci, Elia Abbafati, Claudio Serratore and Michaela Caricato [17], provides a systematic review of SOC among nurses. They report that low SOC is a predictor of depressive state, burnout, and job dissatisfaction among female nurses and that, therefore, SOC could be a health-promoting resource.

Shir Daphna-Tekoah, Talia Megadasi Brikman, Eric Scheier and Uri Balla [18], close the section on health professions with a timely manuscript on COVID-19. They used a unique methodology involving a listening guide and narrative analysis to understand the physical and psychological needs of heath professional during the pandemic, in order to build and provide suitable support programs for those professionals.

Jeremy Mitonga-Monga and Claude-Hélène Mayer [19], present empirical research findings on coping, SOC, burnout, and work engagement in the context of the Democratic Republic of Congo (DRC), where a void in research on salutogenesis and coping still exists. The authors examined the moderating effect of coping in the relationships between SOC, burnout, and work engagement and found that there is a positive relationship between coping and SOC; however, SOC is negatively related to work engagement and burnout. The authors provide recommendations for future theory and practice to increase engagement, performance, and productivity based on increased SOC and coping mechanisms.

International leaders need new skills in the rapidly changing world of work, as well as new resources to cope with and manage stress. In the last article in this issue, Claude-Hélène Mayer and Rudolph M. Oosthuizen [20], present findings from an international study showing that SOC, compassionate love, and coping interrelate are important resources for staying healthy.

## 4. Conclusions

### The Way Forward

This Special Issue presents the latest studies on salutogenesis and coping in specific cultural and transcultural contexts. These studies present particular insights into specific socio-cultural contexts from qualitative and quantitative empirical, theoretical, and conceptual stances. The articles will lead to deeper discourse, new critical thinking, and expanded contextual knowledge, and will build a foundation for future research and applied interventions with regard to salutogenesis and coping.

The studies in the current Special Issue set the ground for continuing research toward even more comprehensive theoretical grounds: studies which incorporate several theoretical backgrounds and explore a broad theoretical model that may help us to understand successful adaptation in various contexts. We suggest that future studies in the field should incorporate several theories into one model: theories of stress appraisal and coping [1], salutogenesis [4,7], and acculturation [21,22], which are fundamental to the understanding of successful adaptation in various situations. Each of these theoretical foundations will contribute its own driven variables to a model that will encompass the socio-ecological surroundings of the participants. Such studies will enable examination of how different demographic and contextual variables, cognitive appraisals, coping resources, and coping and acculturation strategies relate to each other and to psychological adaptation, on one hand, and various psychological problems, on the other. A comprehensive and coherent model of relations among the variables that relies on the above-mentioned three well-established theories could advance our theoretical and practical knowledge of how people cope and adapt in various contexts and cultures. In summary, the results of studies that incorporate these theories may promote the understanding of the effect of coping resources, and strategies, in addition to acculturation strategies (among minority groups) for positive adaptation. Practically, such research has the potential to help parents, educators, leaders, and policymakers to become better aware of the difficulties experienced by individuals who are confronted with meaningful challenges and stressors. This awareness can assist the establishment of research-based, theory-driven prevention and intervention programs to promote adjustment and adaptation in numerous contexts and cultures.
